# Proton affinities and ion enthalpies*

**DOI:** 10.1177/1469066717728451

**Published:** 2017-08-30

**Authors:** John L Holmes, Nick A van Huizen, Peter C Burgers

**Affiliations:** 1 Department of Chemistry and Biological Sciences, University of Ottawa, Ottawa, Canada; 2 Department of Neurology, Laboratory of Neuro-Oncology, Erasmus Medical Center, Rotterdam, the Netherlands; 3 Department of Surgery, Erasmus Medical Center, 3015 CN, Rotterdam, the Netherlands

**Keywords:** Proton affinity, kinetic method, ionisation energy, core-electron ionisation, ion stabilisation, tandem mass spectrometry, gas-phase ion chemistry

## Abstract

Proton affinities of a number of alkyl acetates (CH_3_–C(=O)–OR) and of methyl alkanoates (R–C(=O)–OCH_3_, R=H, alkyl) have been assembled from the literature or measured using the kinetic method. It was observed that the proton affinities for the isomeric species CH_3_–C(=O)–OR and R–C(=O)–OCH_3_ are almost identical, an unexpected result as the charge in these protonated ester molecules is largely at the keto carbon atom and so this site should be more sensitive to alkyl substitution. Analysis of the data, including those from lone pair ionisation and core-electron ionisation experiments available from the literature, indicate that after protonation, extensive charge relaxation (or polarisation) takes place (as is also the case, according to the literature, after core-electron ionisation). By contrast, after lone pair ionisation, which results in radical cations, such relaxation processes are relatively less extensive. As a consequence, changes in ion enthalpies of these protonated molecules follow more closely the changes in neutral enthalpies, compared with changes in enthalpies of the corresponding radical cations, formed by electron detachment. Preliminary analyses of published energetic data indicate that the above finding for organic esters may well be another example of a more general phenomenon.

## Introduction

The ionisation energy (IE) of a molecule M is given by

(1) $$\text{IE}(\text{M})=\Delta_{\text{f}}{\text{H}}^{0}({\text{M}}^{+•})-\Delta_{\text{f}}{\text{H}}^{0}(\text{M})$$

From appropriate IE measurements, the enthalpies of formation of M^+•^, Δ_f_H^0^(M^+•^), may be assessed.

The proton affinity (PA) of the molecule is given by

(2) $$\text{PA}(\text{M})=\Delta_{\text{f}}{\text{H}}^{0}(\text{M})+\Delta_{\text{f}}{\text{H}}^{0}({\text{H}}^{+})-\Delta_{\text{f}}{\text{H}}^{0}({\text{MH}}^{+})$$

From PA measurements Δ_f_H^0^(MH^+^) may be obtained.

The gas phase enthalpies of formation of a significant number of organic cations (also called ion enthalpies) have been determined from IE and PA measurements and also by appropriate appearance energy (AE) determinations.^1^ Thus for example, Δ_f_H^0^ [t-C_4_H_9_]^+^ has been derived from the IE of the t-butyl radical, the PA of isobutene and also by computation, leading to Δ_f_H^0^ [C_4_H_9_]^+ ^= 713 ± 3 kJ/mol.^2,3^ Similarly, Δ_f_H^0^ [CH_3_C^+^(OH)CH_3_] has been derived from AE measurements and the PA of acetone, Δ_f_H^0 ^= 499 ± 3 kJ/mol.^4^ Based on such measurements, it has been well established in a number of publications^5–13^ that plots of ΔH_f_ for a series of organic cations, in which an electron releasing group is successively added to the formal charge bearing site, are a simple exponential function of ion size [ln(**n**)], where **n** is the number of atoms in the ion and is commonly assumed to relate directly to ion size. These ΔH_f_ versus ln(**n**) plots have been shown to be linear for ethyl,^12^ methyl,^5–8^ hydroxy,^5,7–9^, methoxy^5,7–9^ and amino^5,7–9^ substitutions at the formal charge site in species such as methane,^5,12^ the methyl cation,^5,7,8,13^ olefinic,^5,7,8,13^ and aromatic hydrocarbons^5,11^ and compounds containing N,^5,7,8,10^ Si, Ge, Sn and Pb.^12^

In addition to providing a method by which thermochemical data may be estimated, such plots provide physicochemical information. For example, from these observations the location of greatest charge density in an ion may be assigned. In many textbooks, the ions ^+^CH_2_OH, CH_3_^+^CHOH and ^+^CH_2_OCH_3_ are displayed as oxonium ions, with the formal charge on oxygen, e.g. CH_2_ = O^+^H. However, the Δ_f_H^0^ values for these ions, 711, 592 and 667 kJ/mol, respectively, show that the stabilising effect of the methyl substituent at carbon in ^+^CH_2_OH is much greater than at oxygen. It follows that the charge density is greater at C than at O.

It has been established, from a comparison of oxygen 1s core ionisation energies,^14,15^ that organic acids and esters, R_1_-C(=O)-O-R_2_ (for example the isomers acetic acid and methyl formate), protonate at the keto oxygen. With the formal charge on the keto carbon, e.g. CH_3_–^+^C(OH)_2_ and H–^+^C(OH)–OCH_3_, it is not surprising that the former has a significantly lower Δ_f_H^0^ (314 vs. 391 kJ/mol), because in CH_3_–^+^C(OH)_2_ three electron donating substituents are attached to the formal charge bearing site, compared with two for H–^+^C(OH)–OCH_3_. However, this stabilisation is not reflected in the PAs of acetic acid and methyl formate, which are, within experimental error, the same, PA = 783 ± 1 kJ/mol.^1^ This is also unexpected in that the IEs are different, with acetic acid having the lower IE (10.62 ± 0.02 eV, compared to 10.84 ± 0.02 eV for methyl formate) and consistent with the formal charge at the keto group in the radical cation. Indeed, it appears from a survey of the literature (and present work) that the PA of a number of R_1_–C(=O)–O–R_2_ and R_2_–C(=O)–O–R_1_ isomers are remarkably similar, despite the charge of the protonated species being at (the carbon atom of) the keto group.

The purpose of this paper is to establish whether the PAs of R_1_–C(=O)–O–R_2_ and R_2_–C(=O)–O–R_1_ are indeed similar for a wide variety of substituents R, and if so, to provide a rationale. During the course of this work we have compiled and compared the IEs and PAs for a large number of compounds and we have also analysed our data using core-electron ionisation energies available from the literature.

## Experimental

Collision-induced dissociation (CID) experiments were performed using a Bruker Esquire ESI ion trap mass spectrometer as described in Jobst et al.,^16^ van Huizen et al.^17^ and Burgers et al.^18^ The esters were dissolved in methanol at a concentration of 0.01 M. The proton bound ester dimers were generated by infusion of the ester solutions with an acidified (0.1% trifluoroacetic acid) water/methanol (50/50) mixture in a ratio of 10: 190 at an infusion rate of 240 µL h^−1^. The kinetic method^19,20^ was employed to determine relative PA values of the esters. Product ion intensities (R) from proton bound heterodimers were obtained at 50% survival yield^21^ and the obtained lnR values were plotted against the known PA values of reference ester molecules (methyl acetate and octyl acetate^17^) to obtain the new PA values. We^16–18^ and others^22^ have observed that the product ion ratio observed in ion trap experiments depends only slightly on the amplitude and so using low amplitudes (corresponding to high survival yields) does not give more accurate relative PA data, but leads to loss of signal strength only. Further details can be found in Jobst et al.^16^ van Huizen et al.^17^ and Burgers et al.^18^ The D-labelled esters CD_3_C(=O)–O–R and CH_3_C(=O)–O–CD_3_ were prepared by small scale esterification of CD_3_C(=O)–OH with ROH and of CH_3_C(=O)–OH with CD_3_OH, respectively.

## Results and discussion

### The PAs of organic esters

In the following we discuss the PAs of compounds of the type R_1_–C(=O)–O–R_2_ and R_2_–C(=O)–O–R_1_ but have limited ourselves to R_1_ or R_2_ = CH_3_, i.e. we compare the PAs of methyl alkanoates with those of the isomeric alkyl acetates. Since there is ample evidence that the formal charge in protonated organic esters is indeed at the keto carbon atom,^4^ we expected that the PA of R–C(=O)–OCH_3_ would be greater than that of the isomeric compounds CH_3_–C(=O)–OR, following the similar (but opposite) behaviour of their IEs. Surprisingly however, the literature PAs of the isomers acetic acid, CH_3_C(=O)OH, and methyl formate, HC(=O)OCH_3_ are remarkably similar, 783 ± 1 kJ/mol. In a preliminary experiment we generated the proton bound dimer [CD_3_C(=O)OH]•••H^+^•••[HC(=O)OCH_3_] and observed that it dissociated to m/z 64 ([CD_3_C(OH)_2_]^+^) and to m/z 61 ([HC(OH)OCH_3_]^+^) in a ratio of 2.12 ± 0.20. It has been established that in such kinetic method experiments for these classes of compounds, the equation ΔPA (kJ/mol) = 2lnR holds,^16–18^ where R is the ratio of the product ions, in this case 2.12. This leads to a difference in PAs of acetic acid and methyl formate of only 1.5 ± 0.4 kJ/mol and so the PAs of acetic acid and methyl formate are indeed closely similar. The complete list of our measured PA values for the alkyl acetates and methyl alkanoates is shown in Table 1.

**Table 1.** PAs (kJ/mol) of alkyl acetates and methyl alkanoates.

Alkyl acetates			Methyl alkanoates		
Homologue	PA^a^	PA^b^	PA^a^	PA^b^	Homologue
Formic acid	742.0		742.0		Formic acid
Acetic acid	783.7		782.5		Methyl formate
Methyl acetate	821.6		821.6		Methyl acetate
Ethyl acetate	835.7	833.7 ± 0.4	830.2	832.1 ± 0.4	Methyl propanoate
Propyl acetate	836.6	836.9 ± 0.5	836.4		Methyl butanoate
Butyl acetate		839.0 ± 0.6	839 ± 1	838.9 ± 0.6	Methyl pentanoate
Pentyl acetate		839.8 ± 0.7		840.4 ± 0.7	Methyl hexanoate
Hexyl acetate		840.5 ± 0.7		841.3 ± 0.7	Methyl heptanoate
Heptyl acetate		841.0 ± 0.8	844 ± 1	841.8 ± 0.8	Methyl octanoate
Octyl acetate		841.3 ± 0.8		842.4 ± 0.8	Methyl nonanoate
Nonyl acetate		841.8 ± 0.9		842.9 ± 0.9	Methyl decanoate
Decyl acetate		842.4 ± 0.9		843.4 ± 0.9	Methyl undecanoate
Isopropyl acetate	836.6	841.7 ± 0.7	836.6	838.5 ± 0.7	Methyl isobutyrate
*sec*. butyl acetate		844.5 ± 0.5		842.4 ± 0.5	Methyl 2-methylbutyrate
*iso* butyl acetate		839.0 ± 0.5		839.5 ± 0.5	Methyl isovalerate
*tert*. butyl acetate		845.2 ± 1.4^c^	845.5	843.3 ± 0.6	Methyl pivalate
Cyclohexyl acetate		846.1 ± 0.5	846.2	845.8 ± 0.5	Methyl cyclo C_6_H_11_ (MCC)
Phenyl acetate		837.7 ± 0.5	850.5	843.7 ± 0.5	Methyl benzoate
Vinyl acetate	813.9		825.8	829.3 ± 0.5	Methyl acrylate
Phenyl formate		802.6 ± 0.4	821.1		Benzoic acid

PA: proton affinity.

a From Lias et al.^1^

b This work.

c Using Na^+^ bound dimer, see text.

This table also includes some relevant values for branched alkyl groups and values from the literature.^1^ The first three columns in Table 1 give PA data for the alkyl acetates and the last three columns show the PA data for the isomeric methyl alkanoates (in a given row), for example the PA of hexyl acetate is 840.5 kJ/mol, while that of its isomer, methyl heptanoate, is 841.3 kJ/mol. Some of these observations deserve extra comment. Generally speaking, our PA values compare well with the literature values, see for example the values for propyl acetate, methyl pentanoate and methyl cyclohexanecarboxylate (MCC). However, some other values differ by ±2 kJ/mol or more. For example, our PA for isopropyl acetate (841.7 kJ/mol) is 5.1 kJ/mol larger than the literature value (836.6 kJ/mol).

**Figure 1.**
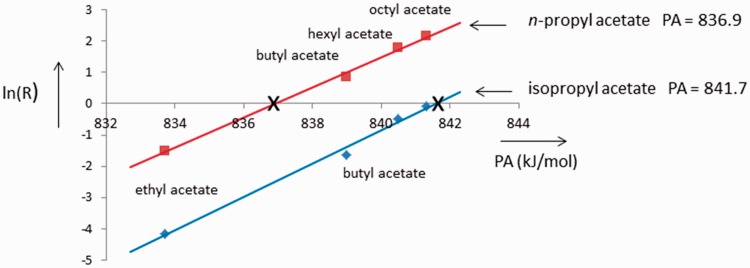
The ln(*R*) values for dissociations of [*n*-propyl acetate•••H^+^•••alkyl acetate] and of [isopropyl acetate•••H^+^•••alkyl acetate] vs. PA. The two curves are shifted by 4.8 kJ/mol. PA: proton affinity.

However, according to the literature (see Table 1), the PA of propyl acetate and isopropyl acetate are the same. This could reasonably be expected, because the charge in the protonated species will be on the carbonyl group, and so branching remote from the charge site will have little effect. Our results for the PA determinations of propyl acetate and isopropyl acetate are given in Figure 1. Here, the PA of both compounds have been measured against the bases ethyl-, butyl-, hexyl- and octyl acetate (see Table 1) and it can be seen that the PA of isopropyl acetate is 4.8 kJ/mol larger than that of propyl acetate. In agreement with our values is the finding that the PA of isopropyl formate (811 kJ/mol)^1^ is 6 kJ/mol larger than the PA of propyl formate (805 kJ/mol).^1^ These matters will be discussed below. Similarly, our derived PA value for methyl benzoate is 6.8 kJ/mol lower than the literature value. In a control experiment, methyl benzoate was measured against MCC and from this result it followed that the PA of methyl benzoate is 2.1 kJ/mol lower than that of MCC, not 4.7 kJ/mol higher. To evaluate the internal consistency of the data presented in Table 1, several such control experiments were performed; for example methyl nonanoate was also measured against methyl 2-methylbutyrate which have the same PAs. The observed intensity ratios R for [(methyl nonanoate) + H^+^]/[(methyl 2-methylbutyrate) + H^+^] formed from the proton bound dimer was found to be 1.1 (lnR = 0.1) leading to ΔPA = (0.2 ± 0.4) kJ/mol.

It was found that the PA of phenyl formate was much lower than all the other ester compounds listed in Table 1, and so the PA of phenyl formate was measured against some nitriles as reference bases (butane-, pentane-, heptane-, nitrile and *tert* butylcyanide^16^).

One particular example deserves further mention. We could not generate protonated *tert* butyl acetate in our ion trap experiments; rather we observed extensive formation of m/z 61, protonated acetic acid. We tentatively conclude that this species is formed by the hydrolysis of transient protonated *tert* butyl acetate by residual water present in the ion trap: CH_3_–^+^C(OH)–OR + H_2_O → CH_3_–^+^C(OH)_2_ + ROH, where R = *t*-butyl; such hydrolysis reactions have been observed previously in ion trap experiments.^23,24^ We have therefore estimated the PA of *tert.* butyl acetate by measuring the product-ion ratio of the sodium bound dimer [*tert.* butyl acetate]•••Na^+^•••[iso propyl acetate] using the lnR versus PA curves for the proton bound and sodium bound dimers as references.^17,18^ Since the slope of the lnR versus PA curve for the sodium bound dimers is only ¼ of the slope of the lnR versus PA curve for the proton bound dimers,^16–18^ the experimental error for the derived PA of *tert* butyl acetate is concomitantly larger, see Table 1.

### PA and methyl group substitutions

It can be seen from Table 1, that except for the isomeric pairs vinyl acetate/methyl acrylate and phenyl formate/benzoic acid, all other paired isomers have very similar PA values (minor, secondary effects, will be discussed later). This means that for saturated and branched R-chains, the PAs of the isomers R-C(=O)–OCH_3_ and CH_3_–C(=O)–OR are almost equal, despite the finding that protonation of organic esters occurs exclusively at the keto group and that the charge is predominantly at the keto C atom.^4^ A confirmation of this result comes from labelling experiments: the labelled proton bound isomeric heterodimers [CD_3_C(=O)OR]•••H^+^••• [RC(=O)OCH_3_] show a ratio for [(CD_3_C(=O)OR) + H^+^]/[(RC(=O)OCH_3_) + H^+^] varying from 2.2 for R = C_2_H_5_ to 0.46 for R = C_10_H_21_, see below, indicating that the PAs of the isomers R–C(=O)–OCH_3_ and CH_3_–C(=O)–OR are equal to within 2 kJ/mol. (Control experiments revealed that isotope effects are negligible; for example, the labelled proton bound dimer of ethyl acetate [CH_3_C(=O)OC_2_H_5_]•••H^+^•••[CD_3_C(=O)OC_2_H_5_] shows a ratio for m/z 92 and m/z 89 of 1.01, whereas the labelled proton bound dimer of methyl propanoate [CH_3_CH_2_C(=O)OCH_3_] •••H^+^•••[CH_3_CH_2_C(=O)OCD_3_] shows a ratio for m/z 92 and m/z 89 of 1.05.)

**Scheme 1.**
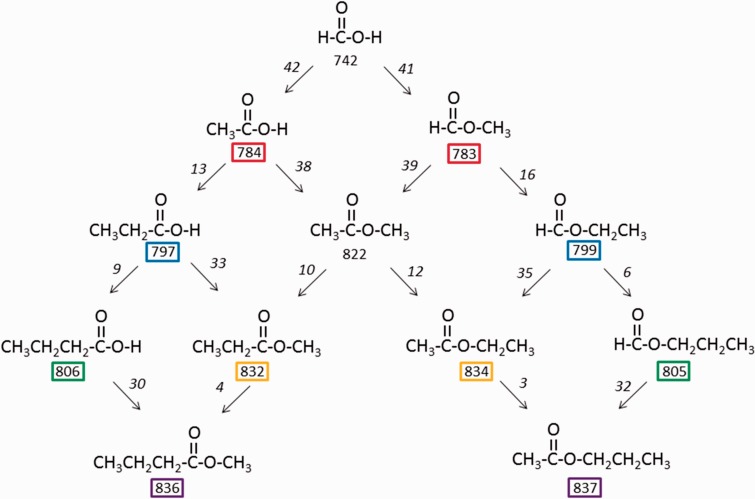
Proton affinities (kJ/mol) of selected organic acids and esters.

The above finding, namely that the PAs of the isomers R–C(=O)–OCH_3_ and CH_3_–C(=O)–OR are almost equal, can be extended to include organic acids and alkyl formates as far as their PAs have been determined. Starting from formic acid as the prototype molecule, we can substitute the C–H hydrogen or the O–H hydrogen by a methyl group and then perform further homologous substitutions. We can then list, for each substitution, the increments in PA, see Scheme 1;^25^ several features emerge, the most obvious being that the PAs of R_1_–C(=O)–O–R_2_ and of R_2_–C(=O)–O–R_1_ are indeed almost equal (including R = H), see the coloured boxes in Scheme 1. For example, starting with formic acid, both C–H and O–H hydrogen substitution by CH_3_ results in an increase in PA of c. 41–42 kJ/mol. Going from acetic acid or methyl formate to methyl acetate again raises the PA by a similar amount (38–39 kJ/mol). These PA increments resulting from methyl substitution cannot be rationalised on the basis of the charge being predominantly at the carbonyl group and clearly another phenomenon is responsible for the observed increments.

### PA, lone pair ionisation (IE) and core-electron ionisation: Inductive and relaxation effects

**Figure 2.**
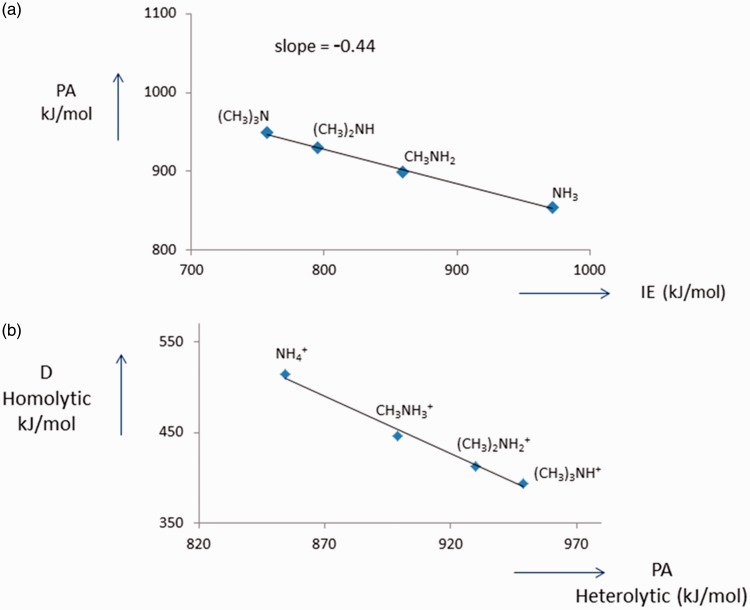
(a) PA as a function IE for the amines NH_3_, CH_3_NH_2_, (CH_3_)_2_NH and (CH_3_)_3 _N. The slope is −0.44. (b) Homolytic bond dissociation energies (D) of the protonated amines as a function of PA. IE: ionisation energy; PA: proton affinity.

It has been shown that a good correlation exists between the PA and IE for a variety of species M.^26–28^ These two quantities are related by

(3) $$\text{PA}(\text{M})=-\text{IE}(\text{M})+\text{IE}({\text{H}}^{•})+\text{D}({\text{MH}}^{+})$$

where IE(H^•^) is the IE of a hydrogen atom and D(MH^+^) is the homolytic bond dissociation energy of MH^+^, MH^+^ → M^+•^ + H^•^ (the PA is the bond dissociation energy MH^+^ → M + H^+^). It is invariably found that a plot of PA versus IE does not yield a slope of −1.0 (as would be expected from equation (3)), but a less negative slope (ca. −0.6).^26–28^ For example, consider successive methyl group substitutions in NH_3_: NH_3_ → CH_3_NH_2_ →(CH_3_)_2_NH → (CH_3_)_3 _N where the plot of PA against IE yields a straight line with a slope of −0.44, see Figure 2(a). According to equation (3), a slope less negative than −1 indicates that the N–H homolytic bond dissociation energy decreases as the PA increases, see Figure 2(b), see also Choi and Boyd.^27^ If a plot of the PA against IE gives a slope of close to −1 (as is the case for the phosphine methyl group substitutions^29^: PH_3_ → CH_3_PH_2_ →(CH_3_)_2_PH → (CH_3_)_3 _P), then the homolytic bond dissociation energy remains constant;^15^ these matters will be discussed in detail elsewhere.

**Figure 3.**
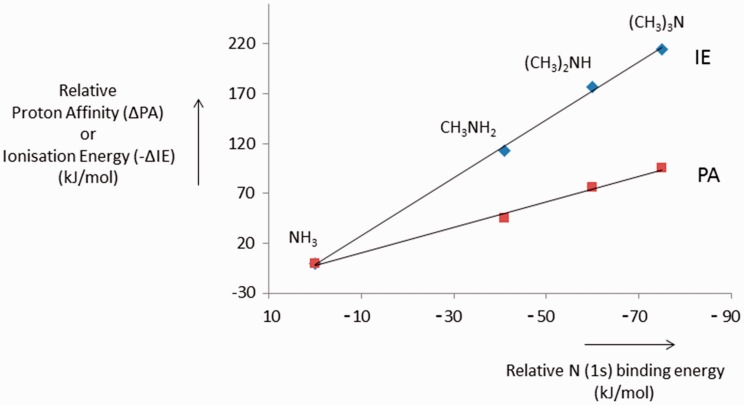
IE and PA as a function of N(1s) ionisation energies for the amines. Values relative to NH_3_ = 0. IE: ionisation energy; PA: proton affinity.

**Figure 4.**
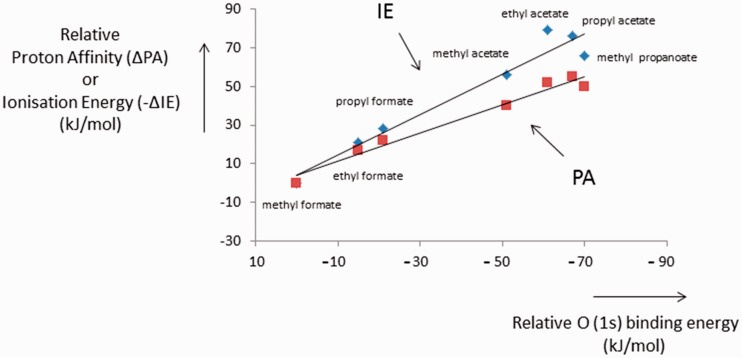
IE and PA as a function of O(1s) ionisation energies for esters. Values relative to methyl formate = 0. IE: ionisation energy; PA: proton affinity.

In addition to the observed correlations between PA and IE, good correlations between PAs and core-electron ionisation energies for certain categories of molecules have also been established;^14,15,30–32^ these have been referred to as the Martin–Shirley correlations.^32^ For example, for alcohols,^30^ it was found that the change in PA from one molecule to another is almost exactly equal to the change in core IE, i.e. a plot of the oxygen 1s IE against the PA gives a slope of −1. It was argued that adding a positive charge to a nucleus (core ionisation) and adding a positive charge adjacent to the nucleus (protonation) will give rise to similar molecular electronic relaxation effects. The proton attachment reaction can be split into two hypothetical steps.^30^ In the first, the proton attaches itself to an atom (for example oxygen) without flow of charge in the molecular framework; shifts in energy of this “reaction” are due to differences in the electron density about the oxygen in the ground state and are inductive effects. In the second hypothetical step, the excess charge is distributed over the whole molecule to minimise Coulombic repulsion (relaxation or polarisation effects). Several groups^30–32^ agree that differences in relaxation energies (rather than differences in inductive effects) dominate both core ionisation and protonation processes. Furthermore, if linear relationships exist between PA and core ionisation energies and between PA and IE, then a linear relation should also exist between IE and core ionisation energies. This is illustrated in Figure 3 for the amines discussed above, in which the PAs and IEs are plotted against the N(1s) binding energies of the amines.^15,30^ (The N(1s) binding energies are averages from Mills et al.^15^ and Martin and Shirley^30^). It can be seen that both IE and PA correlate linearly with the N(1s) binding energy. (Following Mills et al.,^15^ relative binding energies are negative.) However, the slope for the IE curve is significantly larger (2.9) than the slope of the PA curve (1.3) and this parallels the observation that the slope of the PA versus IE curve is significantly less negative than −1, see above. We thus conclude that for this system, the changes in PA are largely governed by relaxation effects, but that changes in IE also reflect changes in inductive effects. Similar plots can be made for other systems, and when such plots are made for organic esters, a remarkable result ensues, see Figure 4, where we show that the curve for PA against oxygen 1s IE has a slope of even less than 1 (0.73), indicating that after protonation, extensive relaxation effects operate and that inductive effects are virtually non-existent. Even for the IE versus oxygen 1s IE curve, the slope is only 1.05 indicating that for the radical cations too, relaxation effects are important. In this respect it should be mentioned that relaxation energies have a tendency to increase with molecular size.^30^ Thus we conclude that for the above organic esters the PA values are indeed largely governed by molecular size, whereas this is less so for the IE values. In such cases, changes in the heat of formation of the protonated species closely follow those of the neutral species and so any conclusions drawn from these PA data as to charge location in the protonated species should be viewed critically.

It can be seen from Table 1 that the PA of methyl acrylate is close to (and slightly *lower* than) that of methyl propanoate. This is in sharp contrast to other α,β unsaturated keto compounds whose PA values are significantly larger (by 10–60 kJ/mol)^25^ than their corresponding saturated analogues. This stabilisation is thought to arise from participation of canonical resonance structures, but clearly such structures are not involved in the case of protonated methyl acrylate. Again, we conclude that the PAs of methyl propanoate and methyl acrylate are primarily determined by size. We also tried to measure the PA of methyl propiolate, HC≡C–C(=O)–O–CH_3_ but this experiment failed: no proton bound dimers could be formed from this compound, it undergoing rapid trimerisation in our ESI experiments to produce m/z 253. The latter ion loses CH_3_OH and 44 Da and so it is most probably not a proton bound trimer, and we propose benzenetricarboxylic acid, trimethyl ester.

### Comparison of ion stabilisation effects from IE and PA measurements

**Figure 5.**
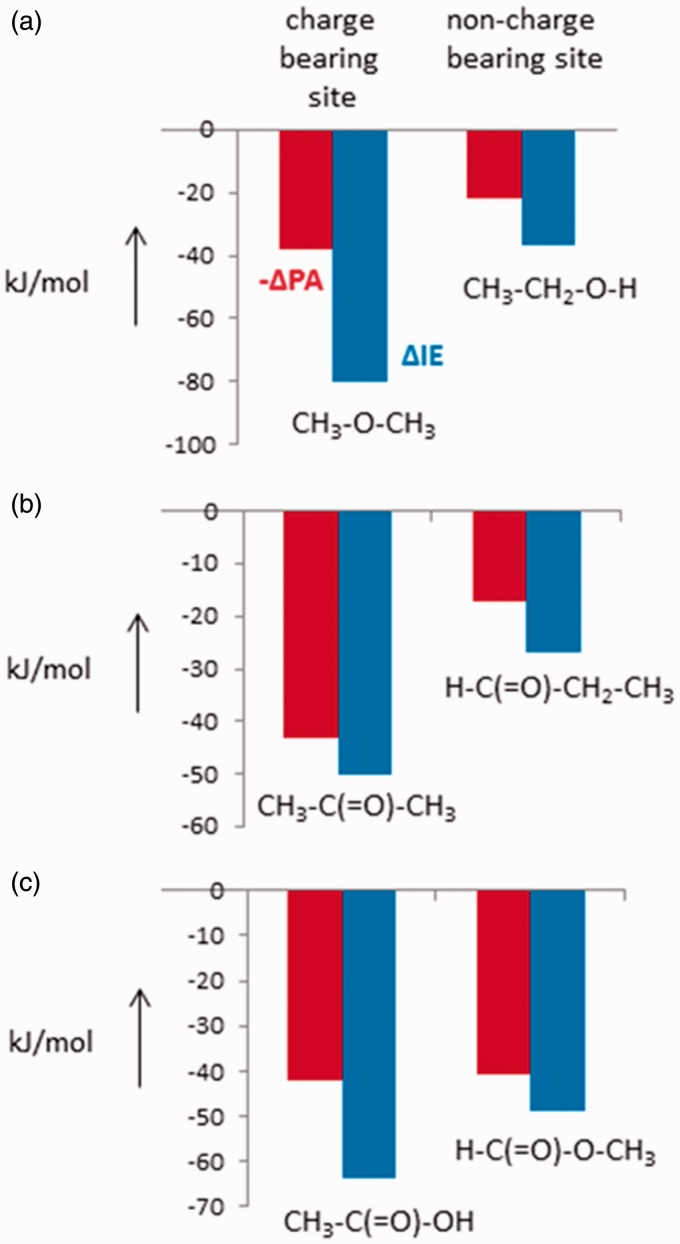
Stabilisation energies as represented by ΔIE (blue bars) or −ΔPA (red bars) for methyl group substitutions at charge bearing and non-charge bearing sites in methanol, acetaldehyde and formic acid. See text for discussion. IE: ionisation energy; PA: proton affinity.

An alternative way to view ion stabilisation effects arising from methyl substitution at a formal charge bearing site versus a non-charge bearing site is presented in Figure 5. Starting from CH_3_OH, we can substitute the O–H hydrogen by a methyl group to yield CH_3_OCH_3_. The stabilisations (measured by a decrease in IE or an increase in PA) are given by the bars, and it can be seen that the increase in PA is about one half the decrease in IE, paralleling the effects discussed above. These stabilisations are those arising from methyl substitution at the charge bearing site. When we substitute the hydrogen at a non-charge bearing site, we find as expected, much smaller stabilisation energies, and again the increase in PA is smaller than the decrease in IE.

A similar situation pertains to ionised acetaldehyde, CH_3_CH = O. Here again, methyl substitution at the charge bearing site (to produce acetone) is associated with larger stabilisation energies (both from IE and PA measurements) than methyl substitution at the non-charge bearing site (to yield propanal), and once more the changes in PA are significantly smaller than the changes in IE.

Similar plots can also be produced for methyl substitutions in formic acid: substitution at the formal charge-bearing site yields acetic acid, while substitution at the non-charge bearing site gives methyl formate, the compounds discussed above. Three features then emerge from Figure 5(c):

(1) The differences in IE are consistent with the formal location of the charge on the keto group, but the effect is relatively small; (2) the differences in IEs and PAs are also relatively small, and (3) there is almost no difference in the PA values, see also above. Such very small differences in PA values could erroneously be taken to show that protonation in organic esters also occurs at the ether oxygen, whereas the cause of this effect lies, see Figure 4, in the dominant relaxation processes occurring after protonation (PA), a process which is of relatively lesser importance after lone pair ionisation (IE). Indeed, according to DFT and MP2 *ab initio* calculations, the positive charge in both protonated methyl formate and protonated acetic acid is largely at the carbonyl C (protonated methyl formate: +0.735 (DFT), +0.923 (MP2); protonated acetic acid: +0.926 (DFT), +1.110 (MP2); Mayer, Personal communication, 2017).

Based on these above findings, we have collected all the IE and PA values available in the NIST website, together with additional data found in the literature. We will report on a detailed analysis of these data in a future publication, but a generalisation (already apparent from the literature) rapidly emerges, namely that for a given category of molecules, the change in PA is usually smaller than the change in IE (with the notable exception of successive methyl substitutions in PH_3_). Thus for a given category of molecules, the changes in ion enthalpies of the protonated species more closely follow those in the enthalpies of the neutral molecules, compared to changes in the ion enthalpies of the radical cations. Another feature of the above *ab initio* calculations is that the incoming proton, once attached to the molecule, retains ca 58% of its charge, with the remainder spread over the other atoms in the molecule. This effect has been observed previously, for example in nitriles,^33^ oxygen containing compounds,^34^ amines^34^ and even amino acids.^35^ Whether this phenomenon also relates to the observation that ion enthalpies of protonated species tend to more closely follow neutral enthalpies (which would appear logical) is also a matter of current investigation.

### Secondary effects upon protonation of organic esters

**Figure 6.**
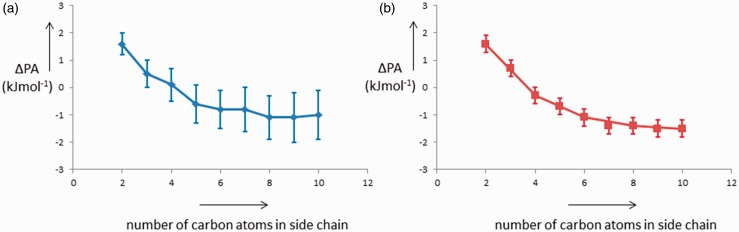
Differences in PA for the isomeric alkyl acetates (CH_3_–C(=O)–OR) and of methyl alkanoates (R–C(=O)–OCH_3_) as a function of R: (a) from laddering experiments, see Table 1; (b) from the isotope labelled dimers [CD_3_C(=O)OR]•••H^+^•••[RC(=O)OCH_3_]. PA: proton affinity.

It can be seen from Table 1 that both phenyl formate and vinyl acetate have lower PAs than their isomers, benzoic acid and methyl acrylate, respectively. Scaled molecular models (for example Dreiding ball-and-stick models) show that the vinyl group in vinyl acetate is very close to the CO function, whereas there is no such interference in the isomer. A similar situation obtains for the phenyl analogues. Also, in protonated vinyl acetate there is a very close proximity between the terminal CH_2_ group and the protonated carbonyl moiety. Such post protonation effects could well affect the stability of the protonated species, but in the absence of more *ab initio* calculations such conclusions must remain speculative. As for secondary effects, it is interesting to note that the PA of ethyl acetate (833.7 kJ/mol) is larger than that of methyl propanoate (832.1 kJ/mol) but with longer alkyl chains the PAs cross (at the pair butyl acetate/methyl pentanoate) to become slightly smaller for the acetates having long chains. This is shown in Figure 6(a), which plots the difference in PA for the isomeric alkyl acetates and methyl alkanoates as a function of chain length, data from Table 1. More precise values for the relative PAs of these isomeric esters can be obtained by performing the kinetic method experiments with isotopically labelled isomers, by observing the product ions from proton bound dimers [CD_3_C(=O)OR]•••H^+^•••[RC(=O)OCH_3_], see Figure 6(b). First, these result again show that the PAs of the isomeric species CH_3_–C(=O)–OR and of R–C(=O)–OCH_3_ are very similar indeed. Second, as the chain gets longer, the PA of the alkyl acetate relative to that of the isomeric methyl alkanoate drops and this behaviour becomes asymptotic. This could indicate that after protonation, some H-bonding takes place between the O–H proton and the hydrocarbon chain, an effect that will be relatively more pronounced in the smaller chain alkyl acetate ions (for example for protonated ethyl acetate such bonding would result in a six-membered ring, but for methyl propanoate, a more strained five-membered ring would ensue). Such effects might also explain why the PA of isopropyl acetate is significantly larger (by 3.2 ± 0.5 kJ/mol) than that of its isomer methyl isobutyrate. Again, in the absence of high level *ab initio* calculations such an interpretation must remain speculative.

## Summary

The PAs of the isomeric alkyl acetates CH_3_–C(=O)–OR and methyl alkanoates R–C(=O)–OCH_3_ were found to be almost identical (to within 2 kJ/mol) for a large number of substituents R. This despite the charge in the protonated species being largely on the carbonyl C atom (as indicated by *ab initio* calculations), and so this site would be expected to be more sensitive to stabilisation effects by alkyl substitution. From a comparison of PA data and core-electron ionisation energies, it is concluded that after protonation of the ester molecules, extensive charge relaxation or polarisation takes place, overwhelming any inductive effects. Thus differences in the heat of formation of these protonated species closely follow differences in heat of formation of the neutral species. Whether the observed behaviour for the organic esters is an (extreme) example of a more general phenomenon is currently being investigated.
